# *trans*,*trans*,*trans*-Diaqua­bis(nicotin­amide-κ*N*)bis­(2-nitro­benzoato-κ*O*)copper(II)

**DOI:** 10.1107/S1600536809007995

**Published:** 2009-03-25

**Authors:** Kou-Lin Zhang, Qiu-Lan Xie, Seik Weng Ng

**Affiliations:** a College of Chemistry and Chemical Engineering, Yangzhou University, Yangzhou 225002, People’s Republic of China; b Department of Chemistry, University of Malaya, 50603, Kuala Lumpur, Malaysia

## Abstract

The water-coordinated metal atom in the title compound, [Cu(C_7_H_4_NO_4_)_2_(C_6_H_6_N_2_O)_2_(H_2_O)_2_], lies on a center of inversion in an all-*trans* octa­hedral environment with slight distortions. The mol­ecule inter­acts with adjacent mol­ecules through O—H⋯O and N—H⋯O hydrogen bonds, forming a layered network parallel to (010).

## Related literature

There are recent examples of diaquadi(aryl­carboxyl­ato)di(nicotinamide)metal(II) compounds, see: Hökelek & Necefoğlu (2007*a* ▶,*b* ▶); Hökelek *et al.* (2007 ▶); Koksharova *et al.* (2006 ▶); Şahin *et al.* (2007*a* ▶, 2007*b* ▶); Stachova *et al.* (2006 ▶); Çaylak *et al.* (2007 ▶); Zhang *et al.* (2009 ▶).
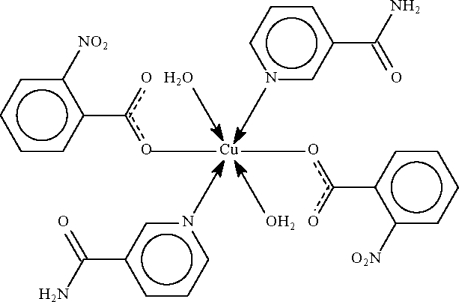


## Experimental

### 

#### Crystal data

[Cu(C_7_H_4_NO_4_)_2_(C_6_H_6_N_2_O)_2_(H_2_O)_2_]
*M* *_r_* = 676.05
Monoclinic, 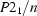

*a* = 7.9582 (3) Å
*b* = 18.7044 (6) Å
*c* = 9.8573 (2) Å
β = 104.012 (2)°
*V* = 1423.63 (8) Å^3^
*Z* = 2
Mo *K*α radiation
μ = 0.84 mm^−1^
*T* = 295 K
0.45 × 0.20 × 0.16 mm

#### Data collection

Bruker SMART area-detector diffractometer
Absorption correction: multi-scan (*SADABS*; Sheldrick, 1996 ▶) *T*_min_ = 0.557, *T*_max_ = 0.877
7295 measured reflections
2507 independent reflections
2069 reflections with *I* > 2σ(*I*)
*R*_int_ = 0.036

#### Refinement

*R*[*F* ^2^ > 2σ(*F* ^2^)] = 0.048
*wR*(*F* ^2^) = 0.111
*S* = 1.12
2507 reflections
229 parameters
4 restraints
H atoms treated by a mixture of independent and constrained refinement
Δρ_max_ = 0.31 e Å^−3^
Δρ_min_ = −0.46 e Å^−3^

### 

Data collection: *SMART* (Bruker, 2000 ▶); cell refinement: *SAINT* (Bruker, 2000 ▶); data reduction: *SAINT*; program(s) used to solve structure: *SHELXS97* (Sheldrick, 2008 ▶); program(s) used to refine structure: *SHELXL97* (Sheldrick, 2008 ▶); molecular graphics: *X-SEED* (Barbour, 2001 ▶); software used to prepare material for publication: *publCIF* (Westrip, 2009 ▶).

## Supplementary Material

Crystal structure: contains datablocks global, I. DOI: 10.1107/S1600536809007995/bt2894sup1.cif

Structure factors: contains datablocks I. DOI: 10.1107/S1600536809007995/bt2894Isup2.hkl

Additional supplementary materials:  crystallographic information; 3D view; checkCIF report

## Figures and Tables

**Table 1:** Selected bond lengths (Å)

Cu1—O1	1.995 (2)
Cu1—N2	2.006 (3)
Cu1—O1*w*	2.537 (3)

**Table 2:** Hydrogen-bond geometry (Å, °)

*D*—H⋯*A*	*D*—H	H⋯*A*	*D*⋯*A*	*D*—H⋯*A*
O1*w*—H11⋯O2^i^	0.85 (4)	1.92 (2)	2.726 (4)	159 (4)
O1*w*—H12⋯O5^ii^	0.85 (4)	2.11 (2)	2.934 (2)	165 (3)
N3—H32⋯O2^iii^	0.85 (4)	2.15 (2)	2.929 (4)	152 (4)
N3—H31⋯O5^iv^	0.85 (4)	2.11 (2)	2.926 (4)	161 (4)

Symmetry codes: (i) 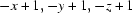; (ii) 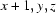; (iii) 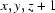; (iv) 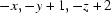.

**Fig. 1.**
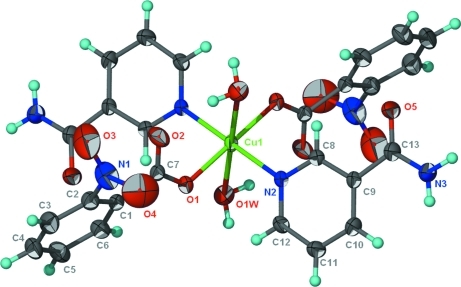
Thermal ellipsoid plot of Cu(H2O)2(C7H4NO4)2(C6H6N2O)2; displacement ellipsoids are drawn at the 50% probabability level, and H atoms as spheres of arbitrary radii.

## supplementary crystallographic information

### Experimental

A water/methanol (1:1 *v*/*v*) solution (3 ml) of copper nitrate trihydrate (0.174 g, 0.6 mmol) was added to a water/methanol (1:1 *v*/*v*) solution (3 ml) of 2-nitrobenzoic acid (0.100 g, 0.6 mmol), sodium hydroxide (0.024 g 0.6 mmol) and nicotinamide (0.073 g, 0.6 mmol). Blue block were obtained after several days (yield: 40%). CH&N elemental analysis: calc. for C_26_H_24_CuN_6_O_12:_ C 46.19, H 3.59,*N* 12.43%; found: C 46.37, H 3.41, N 12.60%.

### Refinement

Carbon-bound H atoms were placed in calculated positions and were allowed to ride on the parent atoms. The oxygen-bound ones were located in a difference Fourier map, and were refined with distance restraints N–H, O–H = 0.85±0.01 Å; an additional H···H 1.39 + 0.01 Å restraint was used. Their displacement parameters were freely refined.

### Crystal data

**Table:**

[Cu(C_7_H_4_NO_4_)_2_(C_6_H_6_N_2_O)_2_(H_2_O)_2_]	*F*(000) = 694
*M**_r_* = 676.05	*D*_x_ = 1.577 Mg m^−^^3^
Monoclinic, *P*2_1_/*n*	Mo *K*α radiation, λ = 0.71073 Å
Hall symbol: -P 2yn	Cell parameters from 3858 reflections
*a* = 7.9582 (3) Å	θ = 2.2–25.0°
*b* = 18.7044 (6) Å	µ = 0.84 mm^−^^1^
*c* = 9.8573 (2) Å	*T* = 295 K
β = 104.012 (2)°	Block, blue
*V* = 1423.63 (8) Å^3^	0.45 × 0.20 × 0.16 mm
*Z* = 2	

### Data collection

**Table:**

Bruker SMART area-detector diffractometer	2507 independent reflections
Radiation source: medium-focus sealed tube	2069 reflections with *I* > 2σ(*I*)
graphite	*R*_int_ = 0.036
φ and ω scans	θ_max_ = 25.0°, θ_min_ = 2.2°
Absorption correction: multi-scan (*SADABS*; Sheldrick, 1996)	*h* = −9→9
*T*_min_ = 0.557, *T*_max_ = 0.877	*k* = −14→22
7295 measured reflections	*l* = −11→11

### Refinement

**Table:**

Refinement on *F*^2^	Primary atom site location: structure-invariant direct methods
Least-squares matrix: full	Secondary atom site location: difference Fourier map
*R*[*F*^2^ > 2σ(*F*^2^)] = 0.048	Hydrogen site location: inferred from neighbouring sites
*wR*(*F*^2^) = 0.111	H atoms treated by a mixture of independent and constrained refinement
*S* = 1.12	*w* = 1/[σ^2^(*F*_o_^2^) + (0.0395*P*)^2^ + 1.8061*P*] where *P* = (*F*_o_^2^ + 2*F*_c_^2^)/3
2507 reflections	(Δ/σ)_max_ = 0.001
229 parameters	Δρ_max_ = 0.31 e Å^−^^3^
4 restraints	Δρ_min_ = −0.46 e Å^−^^3^

### Fractional atomic coordinates and isotropic or equivalent isotropic displacement parameters (Å^2^)

**Table:**

	*x*	*y*	*z*	*U*_iso_*/*U*_eq_	
Cu1	0.5000	0.5000	0.5000	0.02960 (19)	
O1	0.6141 (3)	0.41263 (12)	0.4483 (2)	0.0329 (5)	
O2	0.3896 (3)	0.37179 (15)	0.2856 (3)	0.0531 (7)	
O3	0.3215 (5)	0.2416 (3)	0.0916 (5)	0.1123 (16)	
O4	0.3942 (5)	0.2153 (2)	0.3108 (5)	0.1137 (16)	
O5	0.0632 (4)	0.49124 (18)	0.8331 (3)	0.0642 (9)	
O1W	0.8082 (4)	0.54476 (16)	0.5876 (3)	0.0493 (7)	
H11	0.772 (6)	0.5753 (19)	0.637 (4)	0.067 (15)*	
H12	0.883 (5)	0.522 (2)	0.647 (4)	0.073 (16)*	
N1	0.4256 (5)	0.2375 (2)	0.2043 (5)	0.0624 (10)	
N2	0.4917 (3)	0.45491 (14)	0.6829 (3)	0.0290 (6)	
N3	0.1614 (5)	0.4274 (2)	1.0283 (3)	0.0539 (9)	
H31	0.081 (4)	0.442 (2)	1.065 (4)	0.069 (14)*	
H32	0.241 (4)	0.403 (2)	1.082 (4)	0.075 (16)*	
C1	0.6639 (4)	0.32322 (17)	0.2924 (3)	0.0297 (7)	
C2	0.6033 (5)	0.26281 (19)	0.2137 (4)	0.0372 (8)	
C3	0.7038 (6)	0.2231 (2)	0.1461 (4)	0.0496 (10)	
H3	0.6578	0.1835	0.0929	0.057 (12)*	
C4	0.8740 (6)	0.2428 (2)	0.1585 (4)	0.0552 (11)	
H4	0.9433	0.2170	0.1125	0.065 (13)*	
C5	0.9406 (5)	0.3009 (2)	0.2392 (4)	0.0503 (10)	
H5	1.0561	0.3135	0.2495	0.067 (14)*	
C6	0.8366 (4)	0.3409 (2)	0.3053 (4)	0.0395 (8)	
H6	0.8834	0.3801	0.3591	0.038 (10)*	
C7	0.5449 (4)	0.37176 (17)	0.3480 (3)	0.0289 (7)	
C8	0.3508 (4)	0.46333 (17)	0.7327 (3)	0.0306 (7)	
H8	0.2571	0.4882	0.6785	0.029 (9)*	
C9	0.3378 (4)	0.43678 (18)	0.8613 (3)	0.0304 (7)	
C10	0.4781 (5)	0.3999 (2)	0.9403 (4)	0.0434 (9)	
H10	0.4747	0.3819	1.0276	0.048 (11)*	
C11	0.6240 (5)	0.3899 (2)	0.8889 (4)	0.0461 (10)	
H11A	0.7186	0.3644	0.9402	0.062 (13)*	
C12	0.6259 (4)	0.41833 (19)	0.7608 (4)	0.0364 (8)	
H12A	0.7241	0.4120	0.7268	0.032 (9)*	
C13	0.1760 (5)	0.4530 (2)	0.9067 (4)	0.0419 (9)	

### Atomic displacement parameters (Å^2^)

**Table:**

	*U*^11^	*U*^22^	*U*^33^	*U*^12^	*U*^13^	*U*^23^
Cu1	0.0375 (3)	0.0296 (3)	0.0256 (3)	0.0041 (3)	0.0153 (2)	0.0014 (2)
O1	0.0378 (13)	0.0332 (12)	0.0292 (12)	0.0057 (10)	0.0110 (10)	−0.0034 (10)
O2	0.0318 (14)	0.0677 (19)	0.0557 (17)	0.0093 (13)	0.0028 (12)	−0.0203 (14)
O3	0.061 (2)	0.178 (5)	0.091 (3)	−0.027 (3)	0.006 (2)	−0.071 (3)
O4	0.094 (3)	0.118 (4)	0.140 (4)	−0.030 (3)	0.049 (3)	0.048 (3)
O5	0.0489 (16)	0.107 (3)	0.0436 (15)	0.0375 (17)	0.0254 (13)	0.0285 (16)
O1W	0.0454 (16)	0.0586 (19)	0.0407 (16)	0.0032 (14)	0.0045 (13)	−0.0056 (14)
N1	0.060 (2)	0.051 (2)	0.079 (3)	−0.0141 (19)	0.023 (2)	−0.022 (2)
N2	0.0302 (14)	0.0338 (15)	0.0250 (14)	0.0037 (12)	0.0104 (11)	−0.0004 (12)
N3	0.046 (2)	0.084 (3)	0.039 (2)	0.020 (2)	0.0237 (17)	0.0225 (18)
C1	0.0332 (18)	0.0310 (17)	0.0259 (17)	0.0033 (14)	0.0089 (14)	0.0021 (14)
C2	0.040 (2)	0.0354 (19)	0.037 (2)	0.0021 (16)	0.0110 (16)	0.0003 (16)
C3	0.066 (3)	0.037 (2)	0.046 (2)	0.007 (2)	0.014 (2)	−0.0094 (18)
C4	0.069 (3)	0.054 (3)	0.050 (3)	0.025 (2)	0.027 (2)	0.003 (2)
C5	0.041 (2)	0.052 (2)	0.062 (3)	0.0077 (19)	0.0224 (19)	0.005 (2)
C6	0.036 (2)	0.037 (2)	0.045 (2)	0.0019 (16)	0.0094 (16)	−0.0030 (17)
C7	0.0341 (18)	0.0287 (17)	0.0254 (17)	0.0025 (14)	0.0104 (14)	0.0028 (14)
C8	0.0326 (18)	0.0327 (18)	0.0270 (17)	0.0055 (15)	0.0082 (14)	0.0006 (14)
C9	0.0335 (18)	0.0333 (18)	0.0269 (17)	0.0033 (14)	0.0123 (14)	0.0022 (14)
C10	0.050 (2)	0.054 (2)	0.0303 (18)	0.0127 (19)	0.0168 (17)	0.0124 (17)
C11	0.043 (2)	0.056 (2)	0.042 (2)	0.0189 (19)	0.0159 (18)	0.0153 (18)
C12	0.0342 (19)	0.041 (2)	0.038 (2)	0.0087 (16)	0.0168 (16)	0.0057 (16)
C13	0.042 (2)	0.053 (2)	0.0347 (19)	0.0079 (18)	0.0167 (17)	0.0081 (17)

### Geometric parameters (Å, °)

**Table:**

Cu1—O1^i^	1.995 (2)	C1—C6	1.389 (5)
Cu1—O1	1.995 (2)	C1—C7	1.507 (4)
Cu1—N2^i^	2.006 (3)	C2—C3	1.377 (5)
Cu1—N2	2.006 (3)	C3—C4	1.380 (6)
Cu1—O1*w*	2.537 (3)	C3—H3	0.9300
O1—C7	1.266 (4)	C4—C5	1.375 (6)
O2—C7	1.240 (4)	C4—H4	0.9300
O3—N1	1.216 (5)	C5—C6	1.389 (5)
O4—N1	1.210 (5)	C5—H5	0.9300
O5—C13	1.237 (4)	C6—H6	0.9300
O1W—H11	0.85 (4)	C8—C9	1.389 (4)
O1W—H12	0.85 (4)	C8—H8	0.9300
N1—C2	1.472 (5)	C9—C10	1.381 (5)
N2—C8	1.337 (4)	C9—C13	1.493 (5)
N2—C12	1.342 (4)	C10—C11	1.387 (5)
N3—C13	1.322 (5)	C10—H10	0.9300
N3—H31	0.85 (4)	C11—C12	1.374 (5)
N3—H32	0.85 (4)	C11—H11A	0.9300
C1—C2	1.389 (5)	C12—H12A	0.9300
			
O1^i^—Cu1—O1	180.000 (1)	C4—C3—H3	120.5
O1^i^—Cu1—N2^i^	90.07 (10)	C5—C4—C3	119.7 (4)
O1—Cu1—N2^i^	89.93 (10)	C5—C4—H4	120.1
O1^i^—Cu1—N2	89.93 (10)	C3—C4—H4	120.1
O1—Cu1—N2	90.07 (10)	C4—C5—C6	120.4 (4)
N2^i^—Cu1—N2	180.00 (14)	C4—C5—H5	119.8
O1^i^—Cu1—O1W	96.04 (9)	C6—C5—H5	119.8
O1—Cu1—O1W	83.96 (9)	C5—C6—C1	121.2 (4)
N2^i^—Cu1—O1W	85.84 (10)	C5—C6—H6	119.4
N2—Cu1—O1W	94.16 (10)	C1—C6—H6	119.4
C7—O1—Cu1	123.7 (2)	O2—C7—O1	125.5 (3)
Cu1—O1W—H11	89 (3)	O2—C7—C1	117.3 (3)
Cu1—O1W—H12	122 (3)	O1—C7—C1	117.1 (3)
H11—O1W—H12	103 (4)	N2—C8—C9	123.2 (3)
O4—N1—O3	125.1 (4)	N2—C8—H8	118.4
O4—N1—C2	116.9 (4)	C9—C8—H8	118.4
O3—N1—C2	117.9 (4)	C10—C9—C8	117.7 (3)
C8—N2—C12	118.1 (3)	C10—C9—C13	124.8 (3)
C8—N2—Cu1	119.6 (2)	C8—C9—C13	117.5 (3)
C12—N2—Cu1	122.3 (2)	C9—C10—C11	119.7 (3)
C13—N3—H31	120 (3)	C9—C10—H10	120.2
C13—N3—H32	123 (3)	C11—C10—H10	120.2
H31—N3—H32	115 (4)	C12—C11—C10	118.7 (3)
C2—C1—C6	116.5 (3)	C12—C11—H11A	120.7
C2—C1—C7	122.0 (3)	C10—C11—H11A	120.7
C6—C1—C7	121.2 (3)	N2—C12—C11	122.6 (3)
C3—C2—C1	123.1 (3)	N2—C12—H12A	118.7
C3—C2—N1	117.2 (3)	C11—C12—H12A	118.7
C1—C2—N1	119.7 (3)	O5—C13—N3	122.1 (3)
C2—C3—C4	119.0 (4)	O5—C13—C9	119.9 (3)
C2—C3—H3	120.5	N3—C13—C9	118.0 (3)
			
N2^i^—Cu1—O1—C7	−65.5 (2)	C2—C1—C6—C5	1.7 (5)
N2—Cu1—O1—C7	114.5 (2)	C7—C1—C6—C5	−171.9 (3)
O1W—Cu1—O1—C7	−151.3 (2)	Cu1—O1—C7—O2	−14.1 (5)
O1^i^—Cu1—N2—C8	40.3 (2)	Cu1—O1—C7—C1	160.6 (2)
O1—Cu1—N2—C8	−139.7 (2)	C2—C1—C7—O2	−24.1 (5)
O1W—Cu1—N2—C8	136.3 (2)	C6—C1—C7—O2	149.2 (3)
O1^i^—Cu1—N2—C12	−137.2 (3)	C2—C1—C7—O1	160.7 (3)
O1—Cu1—N2—C12	42.8 (3)	C6—C1—C7—O1	−26.0 (4)
O1W—Cu1—N2—C12	−41.1 (3)	C12—N2—C8—C9	0.7 (5)
C6—C1—C2—C3	−2.6 (5)	Cu1—N2—C8—C9	−176.9 (2)
C7—C1—C2—C3	171.0 (3)	N2—C8—C9—C10	−0.1 (5)
C6—C1—C2—N1	175.3 (3)	N2—C8—C9—C13	177.3 (3)
C7—C1—C2—N1	−11.1 (5)	C8—C9—C10—C11	−0.8 (6)
O4—N1—C2—C3	111.8 (5)	C13—C9—C10—C11	−178.0 (4)
O3—N1—C2—C3	−69.5 (5)	C9—C10—C11—C12	1.2 (6)
O4—N1—C2—C1	−66.2 (5)	C8—N2—C12—C11	−0.4 (5)
O3—N1—C2—C1	112.5 (4)	Cu1—N2—C12—C11	177.2 (3)
C1—C2—C3—C4	1.3 (6)	C10—C11—C12—N2	−0.6 (6)
N1—C2—C3—C4	−176.6 (4)	C10—C9—C13—O5	174.3 (4)
C2—C3—C4—C5	0.9 (6)	C8—C9—C13—O5	−2.9 (5)
C3—C4—C5—C6	−1.6 (6)	C10—C9—C13—N3	−3.5 (6)
C4—C5—C6—C1	0.3 (6)	C8—C9—C13—N3	179.3 (4)

Symmetry codes: (i) −*x*+1, −*y*+1, −*z*+1.

### Hydrogen-bond geometry (Å, °)

**Table:**

*D*—H···*A*	*D*—H	H···*A*	*D*···*A*	*D*—H···*A*
O1w—H11···O2^i^	0.85 (4)	1.92 (2)	2.726 (4)	159 (4)
O1w—H12···O5^ii^	0.85 (4)	2.11 (2)	2.934 (2)	165 (3)
N3—H32···O2^iii^	0.85 (4)	2.15 (2)	2.929 (4)	152 (4)
N3—H31···O5^iv^	0.85 (4)	2.11 (2)	2.926 (4)	161 (4)

Symmetry codes: (i) −*x*+1, −*y*+1, −*z*+1; (ii) *x*+1, *y*, *z*; (iii) *x*, *y*, *z*+1; (iv) −*x*, −*y*+1, −*z*+2.
